# Impact of supervised physiotherapeutic pelvic floor exercises for treating female stress urinary incontinence

**DOI:** 10.1590/S1516-31802007000500003

**Published:** 2007-09-02

**Authors:** Rodrigo de Aquino Castro, Adriana Lyvio Rotta, Patrícia Diniz dos Santos, Marair Sartori, Manoel João Batista Castello Girão

**Keywords:** Urinary incontinence, Pelvic floor, Physical therapy (Specialty), Exercise, Rehabilitation, Incontinência urinária, Assoalho pélvico, Fisioterapia, Exercício, Reabilitação

## Abstract

**CONTEXT AND OBJECTIVE:**

Urinary incontinence is a public health problem that affects more than 200 million people worldwide. Stress incontinence is the most prevalent type. Pelvic floor muscle exercises have been used for treating it, although there is no consensus regarding their application. The aim of this study was to compare the results from treating female stress urinary incontinence with pelvic floor muscle exercises with or without physiotherapist supervision.

**DESIGN AND SETTING:**

This was a randomized, prospective, controlled trial in the Urogynecology and Vaginal Surgery Sector, Universidade Federal de São Paulo.

**METHODS:**

Forty-four women were randomized to be treated for stress urinary incontinence with pelvic floor exercises for three consecutive months, into two groups: one with and the other without physiotherapist supervision. They were evaluated before and after treatment using a quality-of-life questionnaire, pad test, micturition diary and subjective evaluation. Descriptive analysis was used to evaluate the population. The homogeneity of the two groups was evaluated using the Kruskal-Wallis and Chi-squared tests. The success of the two groups after treatment was evaluated using the Wilcoxon test.

**RESULTS:**

The supervised group showed statistically greater improvement in the pad test, micturition diary and quality of life than did the control group. In the subjective evaluation, only 23.8% of the control group patients were satisfied with their treatment. In the supervised group, 66.8% of patients did not want any other treatment.

**CONCLUSION:**

Supervised pelvic floor muscle exercises presented better results in objective and subjective evaluations than did unsupervised exercises.

## RESUMO

**CONTEXTO E OBJETIVO:**

A incontinência urinária é um problema de saúde pública, afetando mais de 200 milhões de pessoas em todo o mundo, sendo que sua forma mais comum é a de esforço. A cinesioterapia do assoalho pélvico vem sendo utilizada com grande sucesso em seu tratamento, embora não exista consenso de protocolo para sua aplicação. O objetivo foi comparar os resultados do tratamento em mulheres com incontinência urinária de esforço por meio de exercícios de fortalecimento do assoalho pélvico com acompanhamento fisioterapêutico, com os de um grupo sem acompanhamento.

**TIPO DE ESTUDO E LOCAL:**

Estudo randomizado, prospectivo e controlado, realizado no Setor de Uroginecologia e Cirurgia Vaginal da Universidade Federal de São Paulo.

**MÉTODOS:**

Realizou-se estudo randomizado com 44 mulheres para tratamento da incontinência urinária de esforço com cinesioterapia perineal por três meses consecutivos, divididas em um grupo com acompanhamento fisioterapêutico e outro sem acompanhamento. Foram avaliadas, antes e depois do tratamento, pelo diário miccional, “*pad test*”, questionário de qualidade de vida (I-QoL), força muscular perineal, e também por avaliação subjetiva. A análise descritiva foi utilizada para caracterizar a casuística estudada. Para verificar a homogeneidade entre os grupos com relação às variáveis contínuas e categóricas, foram aplicados os testes de Kruskal-Wallis e o Qui-quadrado. Pelo teste pareado de sinais de Wilcoxon, avaliou-se o sucesso terapêutico.

**RESULTADOS:**

Ao término do tratamento, o grupo com acompanhamento teve melhores resultados segundo o “*pad test*”, o diário miccional e o questionário de qualidade de vida, quando comparado ao grupo controle. Quando avaliadas subjetivamente, apenas 23,8% das pacientes do grupo controle referiram satisfação com o tratamento. Já no grupo com acompanhamento fisioterapêutico, 66,8% referiram que não desejavam outro tratamento.

**CONCLUSÃO:**

O acompanhamento fisioterapêutico proporcionou melhores resultados subjetivos e objetivos no tratamento da incontinência urinária de esforço feminina pela cinesioterapia do assoalho pélvico.

## INTRODUCTION

According to the World Health Organization (WHO), urinary incontinence affects more than 200 million people all over the world, and it is considered to be a public health problem.^1^ The incidence of this problem is believed to be underestimated, since many women do not seek treatment because they feel embarrassed about it. Guarisi et al.^2^ observed that only 59% of the female patients presenting urinary incontinence looked for medical help. Stress urinary incontinence (SUI), which is defined as urinary leakage from the external urethral ostium associated with stress or effort, is the most common form of the disorder.^3^

There are several factors involved in the physiopathology of urinary incontinence. Among these are the extra-abdominal topography of the bladder neck, presence of a functionally short urethra, hypoestrogenism and lesions in the sphincter mechanism, pudendal nerve, fascias and muscles of the pelvic floor.^4^

The levator ani, which is the main muscle on the pelvic floor, is composed of 70% type I fibers, responsible for slow contraction, and 30% type II fibers, responsible for fast contraction.^5,6^ In SUI, there is a decrease in the number of fast twitch fibers.^7^ Moreover, it has been observed that aging leads to reductions not only in the numbers of type I and II fibers, but also in their diameter, thus increasing the prevalence of SUI among older women.^5^

Clinical treatment for SUI is becoming more popular because of its excellent results, few collateral effects and decreased surgical and hospital costs.^8^ Perineal exercises are widely recommended, since they do not present any collateral effects.^9^

There is no consensus in the data in the literature regarding the number of repetitions, length of the contractions, or frequency/duration of pelvic floor exercise treatment. Because of the anatomical and functional differences observed among women, the standardization of this therapy is inadequate. Furthermore, approximately 30% of women are unable to contract the muscles in the pelvic floor correctly,^10^ and guidance from a specialized professional may improve the results from this kind of treatment.

## OBJECTIVE

The objective of the present study was to compare the results from treating stress urinary incontinence in women using exercises to strengthen the pelvic floor muscles, with or without the aid of a physiotherapist.

## METHODS

The present study was performed in the Urogynecology and Vaginal Surgery Sector of Universidade Federal de São Paulo – Escola Paulista de Medicina (Unifesp-EPM) from June 2001 to July 2003, after the project had been approved by the Unifesp-EPM Research Ethics Committee.

After reading and signing the free and informed consent statement, 44 women were selected for the study. They presented stress urinary incontinence in accordance with the International Continence Society definition (2002). The diagnosis was confirmed by means of urodynamic testing.

As a criterion for inclusion in the study, urinary leakage needed to have been observed during physical examination. Postmenopausal patients needed to have been on topical hormone replacement therapy for no less than three months. Patients presenting any kind of disorder affecting muscle or nerve tissues, or genital bleeding, pregnancy, urinary tract infection, vulvovaginitis, genital prolapse beyond the hymen, atrophic vaginitis or cardiac pacemakers were excluded from the study. The patients included underwent individual physiotherapeutic evaluation to assess their pelvic floor strength by means of bidigital examination during perineal contraction without the association of gluteal and/or adductor muscles, in accordance with Sampselle et al.^11^

The patients were divided into two groups, in a stratified randomized manner, using a computer-generated random number table.^12^ The patients in both groups were instructed to perform the same sequence of exercises daily, repeated in the orthostatic, sitting and supine positions, for 12 consecutive weeks. The groups were constituted as follows:

- Group A: supervised perineal exercises – 23 patients who performed perineal exercises under guidance from a physiotherapist (twice a week, for 45 minutes).
- Group B: unsupervised perineal exercises (control group) – 21 patients who performed perineal exercises at home with monthly assessment from a physiotherapist.

The sequence of contractions of the pelvic floor was as follows: 10 repetitions of five-second hold contractions with five seconds of recovery; 20 repetitions of two-second hold contractions with two seconds of recovery; 20 repetitions of one-second hold contractions with one second of recovery; and five repetitions of 10-second hold contractions with 10 seconds of recovery followed by 5 repetitions of strong contractions together with a cough, with one-minute intervals between each set. At the beginning of each session, general joint warm-up exercises were performed, and at the end of the session, stretching of the hip, adductors, hamstrings and paravertebral muscles were performed.

Once a month, the same physiotherapist evaluated the pelvic floor muscle strength in both groups, by means of bidigital examination, and classified it from zero to five in accordance with Sampselle et al.^11^ The patients were also asked about any difficulties in performing the exercises. They were then informed about their evolution and encouraged to continue the treatment.

In order to evaluate the treatment results, the following were used before and three months after starting the treatment: seven-day urinary diary (micturition diary), one-hour pad test (considered positive when values greater than two grams were obtained) and the Incontinence Quality-of-Life Questionnaire (I-QoL).^13,14^ The use of these instruments has been suggested by the Standardization Subcommittee of the International Continence Society, i.e. women's observations, quantification of symptoms, clinicians' observations, quality of life, and socioeconomic measurements.^15^

The I-QoL questionnaire is composed of 20 questions evaluating the limitations on human behavior, the psychosocial impact and the social embarrassment that urinary incontinence causes. The responses are given scores of between 1 and 5 points and these are summed and converted into a percentage. The higher the percentage is, the better the quality of life is.^13^

Self-assessment (subjective evaluation) after treatment was also used, to take into consideration the patients' satisfaction with their therapy. The women who were satisfied with it said they did not want any other treatment for incontinence. The women who did not report any improvement, and also those who reported improvements but wanted other treatments, were considered to be unsatisfied.

Data analysis was performed by means of the SAS software, version 8.2. Descriptive and inferential analyses were then carried out. The Mann-Whitney and chi-squared frequency tests were used to assess group homogeneity. Analysis of the variables that measured the therapeutic success in each group, before and after the physiotherapeutic intervention, was performed by means of the Wilcoxon signed-rank test for paired data with a significance level of 0.05.

## RESULTS

The groups were homogenous in relation to race, age, menopausal status, length of time with symptoms, number of pregnancies, number of deliveries, urinary incontinence surgery and body mass index, as demonstrated in Table 1. The pad test, I-QoL and urinary diary results were similar in the two groups before treatment (Table 2). Thus, any difference observed may have been exclusively related to the different treatments used.

**Table 1.** Distribution of the groups before treatment (control and supervised pelvic floor muscle exercises, PFME), according to demographics and clinical characteristics

Variable	Category	Control (n = 21)	Supervised PFME (n = 23)	p-value*
Race	Caucasian	12	14	0.802*
	Non-Caucasian	9	9	
Age (years)	Median	54	56	0.9344†
Menopausal status	No	9	11	0.741*
	Yes	12	12	
Length of time with symptoms (years)	Median	5	5	0.7754†
Number of pregnancies	Median	4	4	0.2641†
Number of vaginal deliveries	Median	3	2	0.9716†
Number of cesarean deliveries	Median	1	0	0.0973†
Surgery	No	17	19	0.999t
	Yes	4	4	
Body mass index (BMI)	Median	25.1	25	0.4881†

* Chi-squared test;

† Mann-Whitney test.

**Table 2.** Comparison of results: incontinence quality-of-life questionnaire (I-QoL), pad test and micturition diary for control and supervised pelvic floor muscle exercises (PFME) groups

Variable	Category (median)	Unsupervised PFME (Control)	Supervised PFME	p value†
I-QoL	Before treatment	82	69	0.3717
(points)	After treatment	79	89	0.0456
	p value*	0.2731	0.0001	
Pad test	Before treatment	24.7	20.1	0.8508
(g)	After treatment	15	3.2	0.0018
	p value*	0.0475	0.0002	
Micturitions	Before treatment	11.0	7.0	0.4939
per day	After treatment	10.0	1.0	0.0002
	p value*	0.0396	≪ 0.0001	

* Paired Wilcoxon test;

† Mann-Whitney test.

There was a significant increase in the quality of life of patients who were supervised, from before the treatment to after three months of treatment. When the groups were compared, it was observed that the patients who performed supervised pelvic floor exercises presented a significantly greater increase in quality of life than did the control group (Table 2).

In the pad test, it was observed that only two women (9.5%) in the control group presented negative results (i.e. urine leakage of not more than two grams). In the supervised exercise group, 11 women (48%) presented negative results. There was a signifi-cant decrease in the weight of the pads after treatment, for both groups. However, in the analysis between the groups, it was observed that the supervised group presented a greater reduction in pad weight than did the control group (Table 2).

Analysis of the urinary diary showed that there was a significant decrease in urine leakage episodes in both groups after three months of treatment. When the groups were compared, this decrease was found to be more significant in the group that was guided by a physiotherapist while exercising (Table 2).

**Figure 1:**
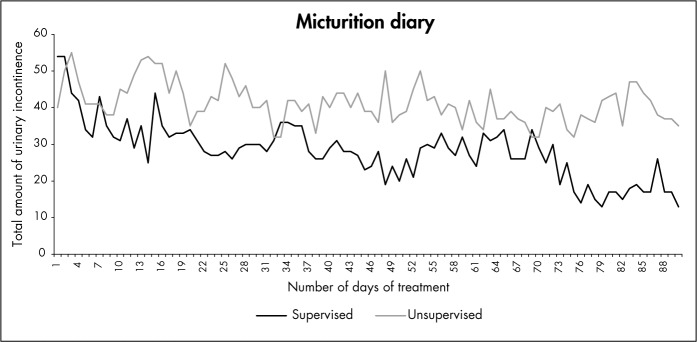
Graphical representation of the total amount of daily urinary incontinence over 90-day period for supervised and unsupervised exercising groups.

Figure 1 shows the variable of urine leakage over the 90 days of treatment. It can be seen that there was a greater decrease in the number of urine leakage episodes in the supervised group.

**Figure 2:**
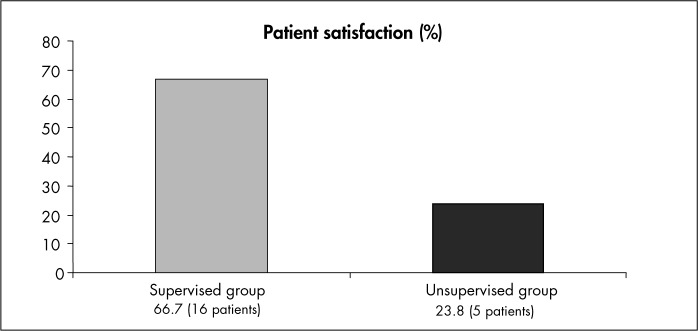
Patient satisfaction according to self-assessment (supervised and unsupervised groups).

Most of the patients who exercised under supervision reported that they did not want any other kind of treatment at the end of the study (they were considered to be satisfied with their treatment). This contrasted with the findings from the self-assessments of the unsupervised group, as shown in Figure 2.

## DISCUSSION

Among the techniques used for treating patients with stress urinary incontinence, pelvic floor exercises are the most common therapy, and these may be indicated as a single treatment or associated with other therapies such as electrotherapy, vaginal cones, biofeedback or bladder training. In some studies, strengthening of the pelvic floor was performed under the supervision of a physiotherapist. Supervision was required because many patients give up exercising when not supervised by a qualified professional.^10,16^

Bo et al.^10^ divided 52 patients presenting SUI into two random groups: one group underwent intensive perineal exercises and the other exercised at home, both for six months. For the first group, the pad test gave a weight of 27 grams before treatment and 7.1 grams after it, while for the at-home exercise group, the weight only decreased from 29.3 to 22 grams.

In the present study, both groups presented better results than those reported by Bo et al.^10^ However, the at-home exercise group also presented a significant improvement. This difference may be due to the fact that, in our study, differing from that of Bo et al.,^10^ all the patients were instructed to perform exactly the same set of exercises.

In 1997, Wong et al.^17^ treated 47 women, placing them randomly in two perineal exercise groups: the intensive group, which visited the clinic twice a week, and the control group, which had only one assessment during the whole four-week treatment. Pad test results of less than or equal to 2 grams were found in 55.3% of the cases, and these were therefore considered to be cured. However, there were no differences between the groups. In our study, with the same criteria, a cure was observed in 9.5% of the control group and in 47.8% of the supervised group.

The results presented by Wong et al.^17^ (1997) may have been influenced by the absence of standardization of the exercises. Those patients were instructed to perform more than 50 contractions a day, which may have led some of them to exercise more than others. Furthermore, because the values prior to treatment were not reported, the importance of the urinary losses in those cases is unknown.

Studies on conservative treatment of urinary incontinence normally do not have a control group. However, as our objective was to evaluate the importance of the physiotherapist for supervising the exercises, the control group underwent the same treatment, except that it was performed at home.

More recently, Sugaya et al.^18^ compared the efficacy of an electronic device used in perineal exercises at home, for two months. In that study, 41 patients were randomly placed into two groups: one that performed exercises for two minutes every time a device sounded (three times a day), and another, the control group (n = 20) wich was instructed to perform the same amount of exercises. Significant improvements were only found in the I-QoL questionnaire. The second pad test and the urinary diary of the control group did not show any improvement. From the control group, only 15% of the patients were satisfied and were aware of any improvement after the treatment. This differed from what was observed in our study, which showed no improvement in I-QoL after the at-home treatment. Moreover, the patients in our study showed themselves to be more satisfied, maybe because of the supervision and monthly assessment.

In the present study, the pad test for the control group presented a significant improvement after treatment when compared with before the treatment. This was a better result than what was obtained by Sugaya et al.,^18^ who did not observe any significant effect.

The urinary diary for the control group showed an improvement at the end of the treatment. This is in agreement with the report from Siu et al.,^19^ who evaluated the results from treatment and participation of women performing exercises at home for four months, with monthly assessments. They observed a significant decrease in daily episodes of urine leakage.

Yoon et al.^20^ studied 44 patients for eight weeks, dividing them into three groups. The first one performed perineal exercises, the second was the control group, and the third performed bladder control. There were no significant differences in pad test results between the three groups. Furthermore, daily and nightly frequency and bladder capacity were unchanged in the group performing the exercises. This may have occurred because of the short period of treatment (eight weeks), which may not have been enough for neural adaptation and muscle hypertrophy.^21^ The present study lasted three months, and there were decreases in daily and nightly frequencies in the supervised group.

All results obtained after treatment, through the pad test, quality of life questionnaire (I-QoL) and urinary diary, showed that the supervised group improved significantly more than the unsupervised group. Similar results for the pad test and urinary diary were reported by Bo et al.,^10^ although their treatment lasted longer (six months). These were in accordance with the conclusions from Hay-Smith and Dumoulin^22^ (2006): the treatment effect might be greater for women who participate in a supervised pelvic floor muscle training program for at least three months.

## CONCLUSION

It was observed that pelvic floor exercises improved stress urinary incontinence. However, for better results, these exercises should be supervised by a physiotherapist.^23^
